# Host genetic control of gut microbiome composition

**DOI:** 10.1007/s00335-021-09884-2

**Published:** 2021-06-22

**Authors:** Jason A. Bubier, Elissa J. Chesler, George M. Weinstock

**Affiliations:** 1 The Jackson Laboratory for Mammalian Genetics, 600 Main Street, Bar Harbor, ME 04609 USA; 2 The Jackson Laboratory for Genomic Medicine, Farmington, CT 06032 USA

## Abstract

The gut microbiome plays a significant role in health and disease, and there is mounting evidence indicating that the microbial composition is regulated in part by host genetics. Heritability estimates for microbial abundance in mice and humans range from (0.05–0.45), indicating that 5–45% of inter-individual variation can be explained by genetics. Through twin studies, genetic association studies, systems genetics, and genome-wide association studies (GWAS), hundreds of specific host genetic loci have been shown to associate with the abundance of discrete gut microbes. Using genetically engineered knock-out mice, at least 30 specific genes have now been validated as having specific effects on the microbiome. The relationships among of host genetics, microbiome composition, and abundance, and disease is now beginning to be unraveled through experiments designed to test causality. The genetic control of disease and its relationship to the microbiome can manifest in multiple ways. First, a genetic variant may directly cause the disease phenotype, resulting in an altered microbiome as a consequence of the disease phenotype. Second, a genetic variant may alter gene expression in the host, which in turn alters the microbiome, producing the disease phenotype. Finally, the genetic variant may alter the microbiome directly, which can result in the disease phenotype. In order to understand the processes that underlie the onset and progression of certain diseases, future research must take into account the relationship among host genetics, microbiome, and disease phenotype, and the resources needed to study these relationships.

**Supplementary Information:**

The online version contains supplementary material available at 10.1007/s00335-021-09884-2.

## Host genetic control of the microbiome impacts health and disease

Candidate genes and genome-wide association studies (GWAS) have yielded significant insight into the genetic variations influencing health and disease. Due in large part to advances in next generation, high-throughput sequencing, and proteomics platforms, the number of reports on the contribution of the gut microbiome to certain diseases has escalated in recent years. The human gut contains 10^13^–10^14^ bacteria from thousands of species, and their collective genomes contain > 150 times more genes than the human or mouse genome (Backhed et al. 2005; Gill et al. 2006; Sender et al. 2016). These bacteria (and viruses, fungi, etc.) are collectively termed the gut microbiome, and their gene content (the metagenome) is often called our second genome (Grice and Segre 2012). Like the host genome, the gut microbiome composition and diversity in each individual are unique (Huse et al. 2012; Zhou et al. 2013).

There is growing awareness in the medical community that an imbalance of the gut microbiome (dysbiosis) is associated with various local and systemic diseases (Shreiner et al. 2015). Dysbiosis has become a hallmark of many diseases, often seen as a symptom of the disease, but not generally considered in the pathogenesis of the disease (Wilkins et al. 2019). Studies using fecal microbiome transplantation (FMT) between obese and lean mice (Turnbaugh et al. 2008) and between lean and obese human donors into mice (Ridaura et al. 2013) indicated that the donor phenotype was transferred to the recipient by the microbiome, demonstrating that the remarkable role commensals play in modulating host phenotype. Since the largest pool of microbes exists in the distal gastrointestinal tract, dysbiosis of the gut microbiome is most readily associated with region-specific gastrointestinal diseases such as Crohn's disease, inflammatory bowel disease (IBD), irritable bowel syndrome (IBS), colorectal cancer, and celiac disease (reviewed in (Gorkiewicz and Moschen 2018)). More recently, attention has turned to the role of the gut microbiome in behavioral and neurological conditions. FMT from a healthy donor to an afflicted patient has been reported to mitigate disease severity in individuals with autism spectrum disorder (Yang et al. 2020) and multiple sclerosis (Engen et al. 2020; Schepici et al. 2019). Other neurological conditions such as Parkinson’s disease (Sampson et al. 2016) and depression (Zheng et al. 2016) have also shown a microbiome-dependent component. Together, these studies demonstrate that the gut microbiome can influence the pathogenesis of not only gastrointestinal diseases, but also behavioral and neurological conditions, likely involving microbial metabolites that function along the gut-brain axis (Cryan et al. 2019).

FMT has become the standard of care for recurrent *Clostridium difficile* infections (Brandt 2012; Wilcox et al. 2020). The clinical benefits of such treatment are only realized if there is successful colonization of donor microbiota in the new host. Some FMT treatments only result in a transient repopulation and may need to be repeated. This transient colonization may be due to host genetic factors preventing successful engraftment. Understanding the host genetic factors that influence microbial engraftment is, thus, essential to establish more practical and cost-effective transplant therapies. Furthermore, the development of therapies that are not dependent on the microbe itself but rather the small-molecule metabolites made by the microbe would be applicable to all hosts.

### Host genetics associated with microbiome composition in humans

Analysis of sequencing data of the human genome completed in 2003 (International Human Genome Sequencing 2004) and the human microbiome in 2016 revealed an association of various diseases with both our human genome and our gut commensals (Human Microbiome Project 2012). These efforts, combined with international programs such as the Metagenomics of the Human Intestinal Tract project (Lee-Sarwar et al. 2020), have provided insight into the crucial host–microbe interactions that function in health and disease. These projects have also contributed numerous bioinformatics tools and reference databases, enhancing our understanding of the specific function of the microbiome in the pathoetiology of disease.

**Fig. 1:**
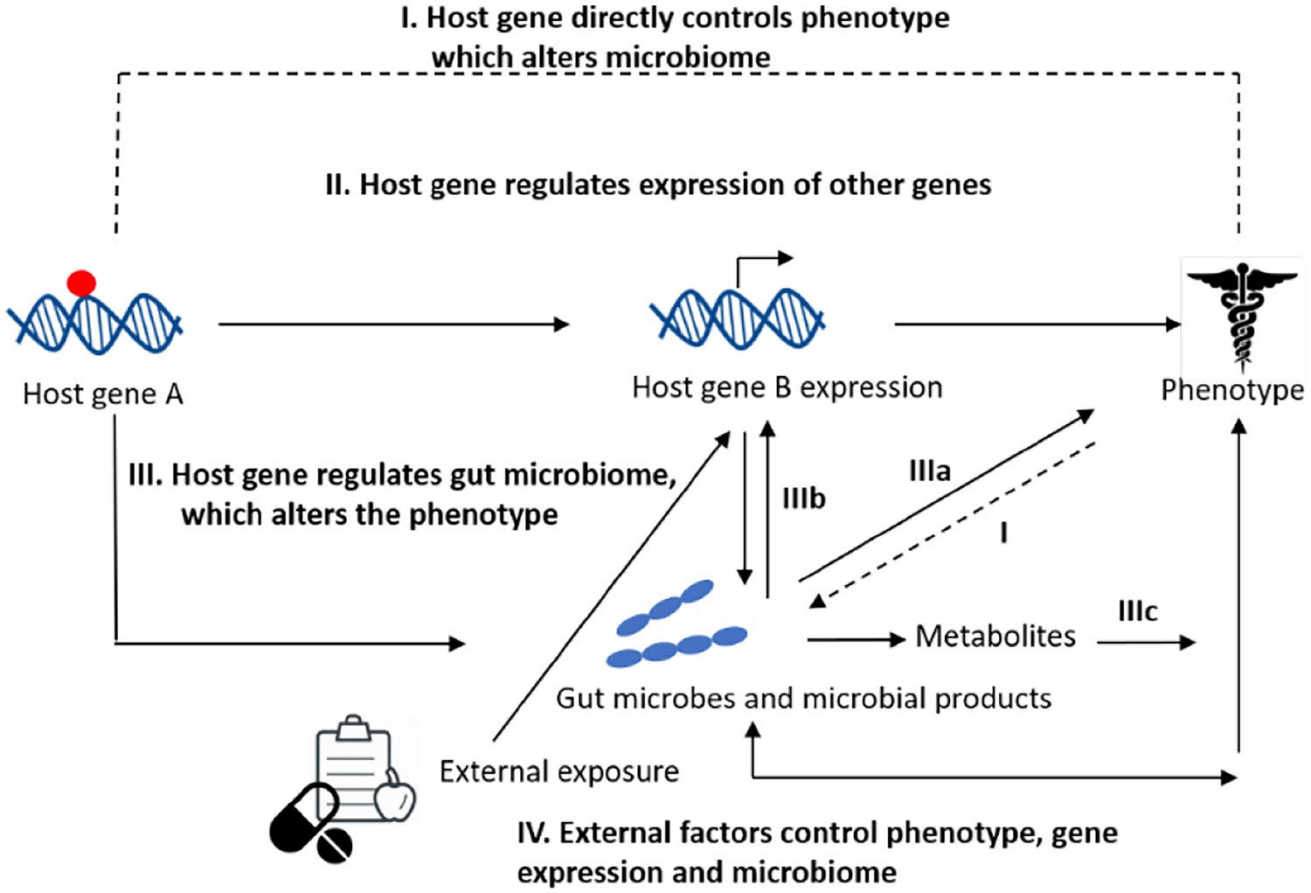
Model representing possible direct and indirect pathways by which the gut microbiome and host genetics control phenotype. I. Individuals with certain gene variants (indicated by red dot) are susceptible to development of an altered phenotype. II. The gene variant modulates the expression of downstream genes and subsequently affects a phenotype, which can alter the microbiome. III. Host genes determine the gut microbiome composition directly. The gut microbiome (IIIa) and their products (such as short-chain fatty acids) can directly modulate the phenotype (IIIc), and/or indirectly affect the phenotype by affecting host gene expression (IIIb). External factors (IV) such as diet or drugs can alter the gut microbiome, leading to a microbiome driven

As outlined in Fig. 1, much has been learned from the human genome as to how a host genetic variant may result in an altered phenotype. The variant may directly (Pathway I) or indirectly (through alterations in the expression of downstream genes for example, Pathway II) modulate a phenotype. The altered phenotype from these two pathways could in turn circle back and modulate the microbiome. Finally, a host genetic variant may directly impact the gut microbiome (Pathway III) which in turn may result in an altered host phenotype either directly through their cell surface molecules (Pathway IIIa), metabolites such as short-chain fatty acids (Pathway IIIc) or indirectly through subsequent effects on host genes (Pathway IIIb).

FMT is the preferred approach for defining cause and effect, but has inherent limitations. Transplantation of numerous recipient mice with the samples from a single human with a specific disease (and a single human control) are underpowered as they represent an *n* = 1 approach (Walter et al. 2020). Further, many studies pool donor samples resulting in an inability to determine what microbiome composition was responsible for the phenotype. Finally, multiple studies fail to verify engraftment, so it is uncertain if failure to alter a phenotype is the true outcome or due to engraftment failure. Future experiments are encouraged to increase rigor to address causality by not pooling donor samples, verifying engraftment of the differential microbiomes (determining if the animals are dysbiotic) and taking other conservative measures to avoid the overstatement of a study’s conclusions (Walter et al. 2020).

The role for the microbiome in host phenotypes is very relevant for discovering genotype–phenotype relations such as in GWAS or other human genetic approaches. The vast majority of this work does not include microbiome analysis and, thus, assumes the mechanisms of Pathways I or II in Fig. 1. A phenotype mediated through the microbiome (Pathway III) presents a different mechanism and, thus, has different approaches to diagnosis and therapy when it is disease related. Moreover, since there is variability in the microbiome between subjects, this can manifest as phenotypic variation, lower penetrance, or other effects that influence human genetic analysis and its use in the clinic. Deeper understanding of microbiome–host genetic relationships is, thus, crucial for medical applications.

A subset of diseases exists for which the genetics of the host determine the microbiome. The most well-studied example is the disorder familial Mediterranean fever (FMF), a genetic autoinflammatory disorder that causes recurrent fevers and painful inflammation in the abdomen, lungs, and joints. FMF is linked to a mutation in the human *MEFV* (Mediterranean fever) gene, which encodes the pyrin protein, a regulator of the innate immune system (Di Ciaula et al. 2020). *MEFV*, through its innate immune function, also controls the gut microbiome composition (Di Ciaula et al. 2020). During times of active FMF, the gut microbiome exhibits a depletion of total numbers of bacteria, loss of diversity, and shifts in relative abundance of populations of *Bacteroidetes*, *Firmicutes*, and *Proteobacteria* phyla (Khachatryan et al. 2008). As the change in microbiome composition occurs during time of active disease symptoms, the pathway of control would be a direct route where the genetic variant impacts the phenotype (i.e., causes MEFV), which results in a subsequent altered microbiome.

### Microbiome composition is a complex heritable trait

Heritability is defined as the fraction of phenotypic variation that can be attributed to a genetic origin. Twin cohort microbiome studies utilizing monozygotic and dizygotic twin cohorts indicate that host genetics control the makeup of the gut microbiome, and that colonization by discrete taxa is highly heritable (Goodrich et al. 2016, 2014; Lim et al. 2017; Turnbaugh et al. 2009; Xie et al. 2016). In a 2014 study of 416 twin pairs, Goodrich et al. (Goodrich et al. 2014) showed using the classic 16S rRNA gene-sequencing approach, in which a region of the 16S rRNA is amplified, sequenced, and compared to databases for taxonomic assignment that 5.3% of the taxa had a heritability greater than 20% (Xie et al. 2016). In 2017, Lim et al. (Lim et al. 2017) showed using the same approach that among 85 taxa, more than half were significantly heritable with heritability ranging between 13.1 and 45.7%, depending on the microbe. A single twin study utilizing a whole-genome shotgun-sequencing approach (Xie et al. 2016) showed strong heritabilities for *Dorea* (42.2%) and *Bifidobacterium* (30.9%) abundance. These significant heritability estimates demonstrate that microbial abundance is amenable to genetic mapping as a complex trait.

### Twin studies identify genomic loci associated with microbial abundance in humans

The twin studies described above were powered well enough to not only calculate heritability for microbial abundance but also identify genomic regions associated with the abundance of specific microbes (Table 1). The most significant findings from the Goodrich studies (Goodrich et al. 2016, 2014) were the association of a SNP (rs2164210) in the lactase gene (*LCT)* with the abundance of *Bifidobacterium* (*p* < 0.001) and a SNP (rs2276731) in the gene for an aldehyde dehydrogenase family member (*ALDH1L1)* with the abundance of unclassified SHA-98 bacteria (*p* < 0.001). In another twin study, a SNP (rs651821) in the apolipoprotein A5 (*APOA5*) gene was associated with the abundance of *Bifidobacterium* in patients with metabolic syndrome (Lim et al. 2017). Twin studies used to calculate the heritability of a trait are most useful for microbiome traits as vertical transmission (mother to offspring) is controlled for in these studies. These twin studies show that specific genomic loci that function in regulating the abundance of discrete gut microbes can be identified by utilizing cohorts of monozygotic vs dizygotic twins to disentangle the shared genetic and environmental factors.

**Table 1:** Human loci associated with microbial abundance

Approach	Chromosome	SNP	Gene	Microbe	Target	Sample size	Reference
Twin Studies	11	rs651821	*APOA5*	*Bifidobacterium*	16S V4	655	(Lim et al. 2017)
Twin Studies	3	rs2276731	*ALDH1L1*	*Unclassified SHA‐98*	16S V4	1126	(Xie et al. 2016)
	2	rs6730157	*RAB3GAP1*	*Bifidobacterium*			
	2	rs2164210	*LCT*	*Bifidobacteria*			
	7	rs1360741	*CD36*	*Blautia*			
	11	rs1506977	*OR6A2*	*Cc 115* (family *Erysipelotrichaceae*)			
	7	rs1182182	*GNA12*	*SMB53* (family *Clostridiaceae*)			
	11	rs1506977	rs1506977	*Cc 115* (family *Erysipelotrichaceae*)			
	7	rs1182182	*GNA12*	*SMB53* (family *Clostridiaceae*)SS			
Genetic association	16	rs2066847, rs2066844, rs2066845,	*NOD2*	depletion of *Bacteroidetes* and *Firmicutes* (particularly Clostridium taxa)	16S V1‐V9	178	(Frank et al. 2011)
	2	rs2241880	*ATG16L1*	depletion of *Bacteroidetes* and *Firmicutes* (particularly Clostridium taxa)	16S V1‐V9		
Genetic association	19	rs601338	*FUT2*	*Alistipes, unclassified Lachnospiraceae, and Coprococcus*	16S V1‐2	47	(Rausch et al. 2011)
Genetic association	19	rs601338	*FUT2*	*Bifidobacteria*	16S V6‐V8	71	(Wacklin et al. 2011)
Genetic Association	6		*HLA‐DRB1*	*Prevotella copri*	16S V1‐V2	114	(Scher et al. 2013)
Genetic Association	16	rs2066844, rs2066845, rs5743277, rs5743293, rs104895431, rs104895467	*NOD2*	*Enterobacteriaceae*	16S V4	474	(Knights et al. 2014)
GWAS	3	rs4894707	*PLD1*	*Akkermansia*	16S V4	114	(Davenport et al. 2015)
GWAS	2	rs56064699	*LCT*	*Bifidobacterium*	16S V3‐5	93	(Blekhman et al. 2015)
	2	rs1050115	*UBXN4*	*Bifidobacterium*			
	3	rs1110168	*PLXND1*	*Prevotella*			
	11	rs1966834	*OR1S1*	*Prevotella*			
	14	rs8019270	*TBPL2*	*Prevotella*			
	20	rs2274669	*PCED1A*	*Alistipes*			
	7	rs10248138	*EPDR1*	*Lachnobacterium*			
GWAS	1	rs12137699	*VANGL1*	Family *Sutterellaceae*	WGS	1514	(Bonder et al. 2016)
	2	rs7605872		Species *Dialister invisus*			
	6	rs4548017		Class *Methanobacteria*			
	9	rs1081306616	*LINGO2*	Genus *Blautia*			
	10	rs1889714		Species *Dialister invisus*			
	11	rs16913594		Species *Bacteroides xylanisolvens*			
	11	rs17115310		Family *Acidaminococcaceae*			
	12	rs10743315		Species *Lachnospiraceae bacterium* 1 1 57FAA			
	21	rs2834288		Family *Oscillospiraceae*			
GWAS (replicated)	2	rs62171178	*UBR3*	*Rikenellaceae*			(Turpin et al. 2016)
	3	rs1394174	*CNTN6*	*Faecalibacterium*			
	1	rs59846192	*DMRTB1*	*Lachnospira*			
	18	rs28473221	*SALL3*	*Eubacterium*			
	4	rs3775467	*MMRN1*	*Weissella*			
	12	chr12:136,228 39:D	*LINC01559* *RNA5SP353* *GRIN2B*	*Weissella*			
	1	rs6666120	*ACTL8*	*Methanobrevibacter*			
GWAS (followed TWIN)	3	rs7433197	*FHIT*	Clostridiales (OTU 181,702)	16S V4	3666	(Beaumont et al 2016; Le Roy et al. 2018)
	6	rs1433723	*TDRG1*	*Clostridiales* (OTU 25,576)			
	1	rs2480677	*ELAVL4*	*Blautia* (OTU 194,733)			
GWAS	1	rs938295	*FBLIM1*	Unclassified *Enterobacteriaceae*	16S V1‐V2	1812	(Wang et al. 2016)
	1	rs75036654	*LINC01137*	Unclassified *Acidaminococcaceae*			
	1	rs597205	*C1orf183*	OTU13305 *Fecalibacterium* Species‐level OTU			
	2	rs4669413	*RP11‐* *521D12.1*	*Blautia* genus			
	2	rs79387448	*SLC9A2*	*Blautia* genus			
	2	rs10928827	*HS6ST1*	*Bacilli *class/*Lactobacillales* order			
	2	rs4621152	*AC007557* *1*	*Gammaproteobacteria* class			
	2	rs56006724	*C2orf83*	Unclassified *Acidaminococcaceae*			
	3	rs11915634	*CNTN6*	*Marinilabiliaceae *family/Unclassified *Marinilabiliaceae*			
	3	rs3925158	*SLC22A13*	OTU10032 unclassified *Enterobacteriaceae *Species‐level OTU			
	3	rs13096731	*FLNB*	*Escherichia Shigella*			
	3	rs59042687	*LINC00879*	*Lactobacillales* order			
	3	rs9831278	*LINC00973*	Unclassified *Marinilabiliaceae*			
	3	rs62295801	*LINC01192*	*Lactobacillales* order			
	3	rs7646786	*LOC344887*	Bacilli class			
	4	rs7656342	*DRD5*	Unclassified *Porphyromonadaceae*			
	4	rs11724031	*SHROOM3*	*Marinilabiliaceae* family			
	4	rs17421787	*RP11‐* *422J15.1*	*Erysipelotrichaceae* family			
	5	rs9291879	*CD180*	Unclassified *Porphyromonadaceae*			
	5	rs249733	*SPRY4*	OTU10032 unclassified *Enterobacteriaceae*			
	7	rs17661843	*ABCA13*	Unclassified *Acidaminococcaceae*			
	8	rs13276516	*TGS1*	OTU10032 unclassified *Enterobacteriaceae*			
	8	rs2318350	*COL22A1*	OTU10032 unclassified *Enterobacteriaceae* Species‐level OTU			
	9	rs17085775	*C9orf71*	OTU10032 unclassified *Enterobacteriaceae*			
	10	rs7083345	*RP11‐* *554I8.2*	*Lactobacillales* order/*Bacilli* class			
	11	rs7113056	*RP11‐* *166D19.1*	*Lactobacillales* order			
	12	rs479105	*PRMT8*	*Bacilli* class			
	12	rs1009634	*AKAP3*	OTU10032 unclassified *Enterobacteriaceae* Species‐level OTU			
	13	rs9300430	*RAP2A*	Gammaproteobacteria class			
	14	rs9323326	*SLC35F4*	*Proteobacteria* phylum			
	14	rs986417	*SIX6*	Unclassified *Acidaminococcaceae*			
	14	rs11626933	*C14orf102*	Unclassified *Erysipelotrichaceae*			
	15	rs12442649	*TMCO5A*	OTU15355 Dialister Species‐level OTU			
	15	rs35275482	*BNIP2*	*Enterobacteriaceae* family			
	16	rs12149695	*FLJ21408*	OTU10032 unclassified *Enterobacteriaceae*			
	16	rs1362404	*TOX3*	*Lactobacillales* order			
	18	rs11877825	*NAPG*	*Erysipelotrichaceae* family			
	19	rs148330122	*SIPA1L3*	*Bacilli* class			
	20	rs2071199	*HNF4A‐AS1*	*Bacilli* class			
	21	rs34613612	*KRTAP8‐1*	*Actinobacteria* class			
GWAS	9	rs150018970	RAPGEF1	*Ruminococcus*	16S V4	3890	(Hughes et al. 2020)
	1	rs561177583		*Coprococcus*			
	16	rs55808472	ARHGAP17	*Butyricicoccus*			
	11	rs4494297	EXT2	*Sutterellaceae*			
	11	rs7118902	SORl1	*Dialister*			
	13	rs35980751	ABCC4	*Porphyromonadaceae*			
	6	rs13207588	FOXP4	*Parabacteroides*			
	2	rs6733298	CCDC85A	*Erysipelotrichaceae*			
	15	rs116865000		*Gammaproteobacteria*			
	9	rs11788336	IKBKAP	*Firmicutes*			
	6	rs34656657	ATXN1	*Firmicutes*			
	4	rs116135844	SPOK3	*Bacteroidales*			
	15	rs117338748	LIPC	*Veillonella*			
GWAS	16	rs3803713	HS3ST4	*Faecalibacterium*	16S V3‐V4	1,068	(Ishida et al. 2020)
	21	rs2839417	C2CD2	*Erysipelotrichaceae*			
	2	rs6545786	2p16.1	*Prevotella*			
	10	rs1033781	10p15.1	*Oscillospira*			
	18	rs885034	18q12.2	Alpha diversity index			
GWAS		rs182549	LCT	*Bifidobacterium*	16S Various	18,340	(Kurilshikov et al. 2021)
	3	rs9864379		*Gastranaerophilales*	V4, V3‐V4, V1‐V2		
	3	rs75754569	IRF1	*Peptococcus*			
	3	rs4428215	FNDC3B	*Oxalobacteraceae*			
	4	rs10805326		*Intestinibacter*			
	4	rs11098863		*Enterorhabdus*			
	7	rs10805326		*Eubacterium coprostanoligenes*			
	9	rs602075	PCK5,RFK, GCNT1	*Allisonella*			
	9	rs736744		Oxalobacter			
	10	rs12781711		*Ruminococcaceae* UCG013			
	10	rs61841503	CUBN	*Peptostreptococcacea e*			
	11	rs10769159		*Ruminococcus1*			
	12	rs12320842		*Faecalibacterium*			
	12	rs11110281		*Streptococcus*			
	13	rs7322849		*Bifidobacterium*			
	14	rs8009993		*Ruminococcaceae* UCG009			
	17	rs7221249		*Erysipelatoclostridium*			
	19	rs67476743		*Tyzzerella3*			
	19	rs830151		*Candidatus* *Soleaferrea*			
	19	rs35866622	FUT2‐FUT1	*Ruminococcus torques*			

### Human genetic association studies or candidate gene studies have associated human genomic regions with microbial abundance

Another approach to determining genomic linkage is through genetic association studies, which test for correlations between altered phenotype and regional genetic variation to identify genomic loci that contribute to the altered phenotype. Genetic association studies have identified several specific relationships between host genetics and microbiome composition (Table 1). The human major histocompatibility complex, specifically the DRB1 haplotype, a rheumatoid arthritis risk locus, is correlated with *Prevotella copri* expansion (*p* < 0.001) (Scher et al. 2013) in untreated new-onset rheumatoid arthritis, but once treated, *Prevotella copri* abundance in chronic patients is not different from healthy controls. The fucosyltransferase 2 (*FUT2)* gene (Rausch et al. 2011; Wacklin et al. 2011), nucleotide-binding oligomerization domain containing 2 (*NOD2)* gene*,* and autophagy-related 16 like 1 (*ATG16L1)* (Frank et al. 2011) were also associated with microbial abundance using genotype association studies.

### Human genome-wide association studies have associated numerous loci with microbial abundance

Genome-wide association studies (GWAS) utilize the approach of genotype association studies but use large sample sizes and unbiased markers throughout the genome to link genotype to phenotype rather than testing the association of a phenotype with only a specific individual gene or genomic region as in genetic association studies. GWAS can link specific SNPs to a phenotype of interest, such as the composition and abundance of specific microbes within the microbiome. They are superior to twin studies as you are not limited to collecting data from just monozygotic and dizygotic twin samples which makes it difficult to reach large sample sizes and the power to detect traits with lower heritabilities. The linked SNP in GWAS may be in a gene or be associated with the nearest gene or genomic feature by convention. As shown in Table 1, GWAS have enabled the detection of numerous SNPs associated with the abundance of specific microbes. Like the twin studies (Xie et al. 2016), GWAS identified *LCT* in association with *Bifidobacterium* (Blekhman et al. 2015). One GWAS confirmed the association of SNPs in ubiquitin-protein ligase E3 component *n*-recognin 3 (*UBR3)* gene, contactin 6 (*CNTN6)* gene, DMRT like family B with proline-rich C-terminal 1(*DMRTB1)* gene*,* and spalt-like transcription factor 3 (*SALL3)* gene that was associated with the abundances of *Rikenellaceae*, *Faecalibacterium*, *Lachnospira,* and *Eubacterium*, respectively (Turpin et al. 2016) in multiple independent cohorts. The most GWAS hits reported to date were found in a study by Wang et al. (Wang et al. 2016) in which 40 different loci were significantly associated with microbial abundance at a variety of levels (class, order, family, or genus) in two cohorts from northern Germany totaling 1812 individuals. Thus far, only one GWAS study used whole-genome shotgun sequencing rather than 16S sequencing to inventory the microbiome composition (Bonder et al. 2016). In this study of 1514 individuals, nine genetic loci were associated with specific microbes classified at levels ranging from the family to the species level. A recent GWAS study utilizing numerous populations and microbiome sequencing methods identified 20 loci and reproduced the *LCT* association with *Bifidobacterium* (Kurilshikov et al. 2021). Overall, twin studies, genetic association studies, and GWAS have identified at least 110 different loci associated with the abundance of specific gut microbes.

The GWAS and genetic association studies approach to calculating heritability in microbiome-related traits must be interpreted with caution (Tam et al. 2019). In these studies, vertical transmission from mother to offspring is not controlled for, unlike in twin studies, and the mode of child delivery also affects the microbiome (Dominguez-Bello et al. 2010). In addition, large, population-based studies such as these do not account for diet or environment, two of the strongest drivers of microbiome composition (Dominguez-Bello et al. 2010; Rothschild et al. 2018). In GWAS, the population size and significance level needed to correct for genome-wide multiple testing and for traits of low heritability that remain under consideration (Dudbridge and Gusnanto 2008). GWAS studies only explain a proportion of the heritability, with other factors such as epistatic and gene-environment interactions not captured (Manolio et al. 2009). Finally, associations between host gene and bacterial abundance do not often replicate across GWAS studies, likely due to variation in diet, environment, and specific population studied.

### Genetic analysis identifies loci associated with microbial abundance in mice

Microbiome studies in laboratory mice allow for the control of many variables within an experiment that are not controllable in human studies. Laboratory mice are an important model system for microbiome studies due to the ability to produce germ-free (GF) or microbiome-depleted animals and introduce microbiomes by FMT or by manipulating the microbiome by other methods such as treatment with antibiotics or altering the diet. This type of control is essential as the environment, and diet has been shown to have the most substantial effect on the microbiome (Dong and Gupta 2019; Rothschild et al. 2018). When mice are provided a defined environment controlling for location, room, diet, and cage effect within a study, host genetics accounts for a large proportion of remaining microbial variation. The genetic effect on the microbiome is illustrated by the intrinsic difference in microbiome between inbred strains of mice (Benson et al. 2010; Campbell et al. 2012; Leamy et al. 2014; McKnite et al. 2012; Org et al. 2015). The combination of variation in the microbiome by strain and the powerful tools of mouse genetics offer a highly effective approach for studying the genetic control of the microbiome.

Quantitative trait loci (QTL) mapping in genetically diverse mice has enabled the identification of genomic regions associated with the microbiome (Table 2, Supplementary Table 1). The first mouse microbial abundance QTL studies, performed in generation four of a C57BL/6 J x ICR advanced-intercross line (AIL) (Benson et al. 2010) using V1-V2 16S sequence from fecal pellets, identified 18 significant or suggestive host QTL, with each QTL accounting for 1.6–9.0% of the variation in microbe abundance. AILs accumulate additional crossovers with every successive generation, leading to a population with smaller linkage disequilibrium (LD) blocks. The original study was followed up four years later using the 10^th^ generation intercross of these mice. This mapping cross identified 42 QTL. Each of the identified QTL explains an average variance of 4.64% of a particular microbe’s microbial abundance. Additional studies have been performed through the years, all utilizing 16S sequencing for microbiome composition in various mouse populations such as the BXD Recombinant Inbred Panel (McKnite et al. 2012; Perez-Munoz et al. 2019), Collaborative Cross (Bubier et al. 2020; Snijders et al. 2016), Hybrid Mouse Diversity Panel (Org et al. 2015; van Opstal and Bordenstein 2015), and Diversity Outbred (DO) mice (Kemis et al. 2019). These studies have contributed an additional 348 loci associated with microbial abundance (Supplementary Table 1). Particularly for mice, there have been few occurrences of the same locus being found in multiple studies. Some of this ‘failure to replicate’ can be attributed not only to the differential diets, husbandry practices, and health status found across studies and facilities, but also to the fact that the same genetic polymorphisms are not present in the same populations and would, thus, not be expected to replicate. For example, there will be loci detected in the BXD RI population that will not be detected in the DO because of the lack of DBA2-specific polymorphisms in the latter population. Taken together, the discovery of so many loci suggests that there are many genes involved in the control of the microbiome, but the inability to replicate genetic loci across studies indicates the importance of testing the causative loci using genetic knock-out experiments.

**Table 2:** Mouse microbial QTL mapping studies

Publication	# Strains	Population	Number of Loci	Statistical criteria	Source	Target
(Benson et al. 2010)	645 animals	G4 AIL (B6J x ICR)	18	Significant or suggestive	Fecal pellets	16S V1‐2
(Hillhouse et al. 2011)	314 animals	F2 (B6 x AJ)	10	Significant	Cecal contents	Helicobacter
(McKnite et al. 2012)	61 animals (30 Strains)	RI (BXD)	9	Significant	Fecal pellets	16S V1‐2
(Leamy et al. 2014)	472 animals	G10 AIL (B6J x ICR)	42	1 significant post FDR	Fecal pellets	16S V1‐2
(Org et al. 2015)	599 (110 Strains)	HDMP	7	Significant	Cecal contents	16S V4
(Wang et al. 2015)	334 animals	F2( WSB/EiJ x PWH/PhJ)	20	Significant	Cecal contents	16S V1‐2
(Snijders et al. 2016)	293 animals (30 Strains)	RI (CC)	169	− log10(P value) > 6)	Fecal pellets	16S V4
(Kemis et al. 2019)	500 animals	Outbred (DO)	28	4 Significant	Fecal pellets	16S V4
(Perez‐Munoz et al. 2019)	~ 128 animals (32 Strains)	RI (BXD)	27	Significant	Cecal contents	16S V5‐V6
(Suzuki et al. 2019)	70 wild animals	Wild Mice	24	− log10(*p* value) > 6)	Cecal contents and fecal pellets	16S V4
(Bubier et al. 2020)	201 animals (108 Strains)	RI (pre‐CC)	18	Significant post FDR	Cecal contents	16S V1‐2, V4

### Gene knock-out studies validate host genes controlling microbiome abundance

A common approach to verifying the involvement of a gene in a process is to inactivate the gene of interest through genetic engineering and define the effect on phenotype. As it relates to microbial abundance, the knock-out (KO) of specific genes has been shown to produce distinct gut microbiomes or altered bacterial colonization engraftment compared to control mice in which the target gene is not inactivated. At least 30 genomic loci have been identified that, when deleted from the germline, result in altered microbiome composition, often in addition to other phenotypes (Table 3). For example, the absence of activation-induced cytidine deaminase (AID) results in the absence of hypermutated IgA. The lack of IgA in these mice makes them susceptible to predominant and persistent expansion of segmented filamentous bacteria (SFB) (Suzuki et al. 2004). In other examples, an intervention may be necessary to reveal an altered microbiome phenotype in KO mice. The altered microbiome composition of NLR family, pyrin domain containing 3 (*Nlrp3)* KO mice (*Nlrp3*^*tm1Bhk*^), is not as apparent until the mice are subjected to environmental manipulation, such as feeding the mice a Western lifestyle diet (Pierantonelli et al. 2017), which exposes the microbiome differences between KO and control mice. The numerous mutations with effects on the microbiome demonstrate the variety of genes through which the host maintains the critical homeostatic regulation of the microbiota.

**Table 3:** Mouse knock-out studies demonstrating altered microbiome composition

Gene	Chromosome	Phenotype	Reference
*Tlr5*	1	The gut microbiotica shows enrichment or reduction of 116 bacterial phylotypes relative to wild-type controls and transplanting gut microbiota from homozygotes to germ-free control hosts confers many aspects of the metabolic disease phenotype	(Chassaing et al. 2014); (Vijay-Kumar et al. 2010)
*Card9*	2	The LEfSe analysis revealed differences including decreases in *Adlercreutzia* (genus), *Actinobacteria* (phylum), and *Lactobacillus* reuter in the Card9 − / − mouse microbiota. Mice fail to metabolize tryptophan into metabolites that act as aryl hydrocarbon receptor (AHR) ligands	(Lamas et al. 2016)
*Pglyrp3*	3	Have reduced *Lactobacillus*/*Lactococcus*, *Enterobacteriaceae,* and *Eubacterium rectale*/*Clostridium coccoides*, and Clostridium perfringen groups	(Saha et al. 2010)
*Pglyrp4*	3	Have reduced *Lactobacillus*/*Lactococcus* and segmented filamentous bacteria groups increased *Bacteroides* group	(Saha et al. 2010)
*Tlr2*	3	Display a threefold increase in Firmicutes and a slight increase in Bacteroidetes compared with controls	(Caricilli et al. 2011)
*Lepr*	4	Have a significant higher abundance of *Firmicutes*, *Proteobacteria*, and *Fibrobacteres phyla* in db/db mice compared to lean mice	(Geurts et al. 2011)
*Ptpn11*	5	Have an increase in Enterobacteriaceae and a decrease in Firmicutes were observed in the colon of these mice	(Coulombe et al. 2016)
*Aicda*	6	Mice exhibit an increase in bacteria, especially anaerobic bacteria, in the small intestine compared with wild-type mice. Some mice exhibit an expansion of unclassified *Lachnospiraceae* of the order *Clostridiales* while others exhibit increased *Bacteroidales* or *Lactobacillus* compared with wild-type mice	(Wei et al. 2011)
*Reg3g*	6	Mice exhibit a higher mucosal bacterial loads (gram-positive Firmicutes phylum [*Lactobacillus*, *Eubacterium rectale*, and segmented filamentous bacteria (SFB) groups]) compared with wild-type mice; however, luminal bacterial loads are normal	(Vaishnava et al. 2011)
*Lep*	6	ob/ob animals have a 50% reduction in the abundance of *Bacteroidetes* and a proportional increase in Firmicutes	(Ley et al. 2005)
*Nlrp2*	7	Dysbiosis marked by increased obesity-associated *Erysipelotrichaceae* but reduced *Lachnospiraceae* family and the associated enzymes r	(Truax et al. 2018)
*Nlrp6*	7	Mice and co-housed wild-type mice exhibit expanded bacterial phylotypes compared with wild-type mice	(Elinav et al. 2011)
*Pglyrp1*	7	Have reduced segmented filamentous bacteria	(Saha et al. 2010)
*Fut2*	7	*Salmonella typhimurium susceptibility*	(Goto et al. 2014)
*Nod2*	8	Relative abundances of several clostridial genera were associated with disease phenotype, NOD2 composite genotype, and/or ATG16L1genotype	(Frank et al. 2011; Rehman et al. 2011)
*Myd88*	9	Increased abundances of *Lactobacillaceae*, *Rikenellaceae,* and *Porphyromonadaceae phylotype*	(Wen et al. 2008)
*Apoa1*	9	12% variation of a Partial Least-Square Discriminate Analysis of microbiota structure accounted for by genotype	(Zhang et al. 2010)
*Mmp7*	9	Increased sensitivity to *Salmonella typhimuriu*m. KO mice also have increase *Firmicutes* (specifically *Clostridia*) an decreased *Bacteroidete*) compared to wild type	(Wilson et al. 1999), (Salzman et al. 2010)
*Atg5*	10	Have a dramatically altered composition of the gut microbiota and reduced alpha diversity. “*Candidatus Arthromitus*” and the *Pasteurellaceae* family were increased in KO mice, whereas *Akkermansia muciniphila* and the *Lachnospiraceae* family were reduced	(Yang et al. 2018)
*Ikzf1*	11	The intestinal flora contains more numbers and more diverse groups of bacteria than in controls	(Georgopoulos et al 1994)
*Nlrp3*	11	Mice fed a Western diet show a greater gut microbiota dysbiosis than controls on the same diet	(Pierantonelli et al 2017)
*Pik3cg*	12	Between WT and KO 11 taxa were found to increase significantly and five taxa decreased significantly in KO mice compared to WT mice	(Li et al. 2020)
*Igha*	12	Mice deficient in IgA harbor an increased abundance of SFB	(Suzuki et al. 2004)
*Ccl28*	13	The abundance of Class *Bacilli* bacteria is increased in the intestine	(Matsuo et al. 2018)
*Sugct*	13	Mice show differences in the proportion of and type of bacteria species in stool, with an increase of firmicutes relative to Bacteroidetes (strongest in Blautia genus containing the families Ruminococcaceae and Lachnospiraceae, then Adlercreutzia genus, Bilophilia genus, and AF12 genus and a decrease in Bifidobacterium genus); microbiome changes resemble those seen in microbiome disbalance in metabolic diseases like diabetes	(Niska-Blakie et al. 2020)
*Olfm4*	14	Following oral challenge, mice exhibit reduced colonization by *Helicobacter pylori* and increased infiltration of inflammatory cells in the gastric mucosa compared with wild-type mice	(Liu et al. 2010)
*Vdr*	15	Lactobacillus was depleted in the fecal stool, whereas Clostridium and Bacteroides were enriched. Bacterial taxa along the Sphingobacteria-to-Sphingobacteriaceae lineage were enriched	(Jin et al. 2015)
*Retnlb*	16	Fifteen Bacteriodetes lineages, and 1 lineage of Proteobacteria, changed in abundance between genotypes, whereas 15 Firmicutes lineages changed in abundance	(Hildebrandt et al. 2009)
*Percc1*	17	Display an altered intestinal and fecal microbiome composition	(Oz-Levi et al. 2019)
*Pglyrp2*	17	Have reduced Lactobacillus/Lactococcus, segmented filamentous bacteria, Clostridium perfringens, and Bacteroides groups	(Saha et al. 2010)
*Npc1*	18	The gut microbiota composition shifted and increased microbial richness and diversity Specifically, Staphylococcus spp. and unclassified Mogibacteriaceae spp. Mice showed significantly higher levels in relative abundance in the KO mice compared to WT mice, whereas the abundance of Allobaculum spp. was significantly lower. Relevantly, the unclassified Mogibacteriaceae spp	(Houben et al. 2019)
HLA-DRB1*0401	Tg	Clostridium-like bacterium abundance altered	(Gomez et al. 2012)
DEFA5	Tg	Mice have a decreased proportion of bacteria from the Firmicutes, and decreased SFB	(Salzman et al. 2010)

Conditional deletion of toll-like receptor 5 (*Tlr5)* from intestinal epithelial cells shows low-grade inflammation, metabolic syndrome, and colitis as compared to wild-type littermates (Chassaing et al. 2014). These conditional KO mice show enrichment or reduction of 116 bacterial phylotypes relative to controls. Antibiotic treatment of the conditional KO mice eliminates the inflammation and associated metabolic syndrome (Chassaing et al. 2014). Transplanting gut microbiota from homozygote conditional KO mice to germ-free control hosts confers many aspects of the metabolic disease phenotype on those mice (Vijay-Kumar et al. 2010). These studies specifically showcase the importance of intestinal *Tlr5* in the maintenance of the gut microbiome.

Another approach using genetically engineered mice to dissect host control of the microbiome has been to produce mice that express human genes from a transgene. To understand the role of antimicrobial peptides in microbiome composition, the human alpha-defensin (DEFA5) gene, a component of enteric mucosal innate immunity, was introduced into FVB mice (Salzman et al. 2010). The transgenic expression of DEFA5 resulted in mice with a decreased proportion of Firmicutes and decreased SFB colonization compared to non-transgenic control FVB mice. This manipulation suggests that alpha-defensins play an essential role in regulating the makeup of the commensal microbiota. The creation of genetically identical mouse strains that differ in the presence of one gene and that display significant differences in microbiome composition supports the concept that the host genotype controls the microbiome and may subsequently affect disease phenotypes.

### Cross-species conservation of host genes that modulate the microbiome

Many confounding factors, especially diet and environment which are strong microbiome composition drivers, prevent replication across studies in humans and mice. Despite these challenges, some loci have demonstrated conserved function across species. For example, the genes *NOD2* (Frank et al. 2011; Knights et al. 2014; Rehman et al. 2011) and *FUT2* (Goto et al. 2014; Rausch et al. 2011) have been associated with microbiome composition in both species. *NOD2* was identified in a human GWAS as a host gene associated with the microbiome composition and inflammatory bowel disease. This dysbiosis was recapitulated in KO mice revealing alteration in multiple distinct microbes associated with the disease phenotype. *FUT2*, the gene responsible for the ABO histo-blood group antigens, was associated in human studies with Crohn's disease and altered microbial community composition. *Fut2* knock-out mice result in altered epithelial fucosylation and increased susceptibility to *Salmonella typhimurium.* These studies implicate conserved functions across species for *NOD2* and *FUT2* in modulating the gut microbiome.

### Microbiome control of host gene expression

GF mice display an array of physiological and behavioral abnormalities (Clarke et al. 2013; Desbonnet et al. 2014; Diaz Heijtz et al. 2011; Neufeld et al. 2011). Because they lack a normal microbiota, the epithelial barrier function, gut homeostasis, and innate and adaptive immune functions of GF mice develop differently (Hooper and Gordon 2001; Lundin et al. 2008). These developmental differences result in altered hippocampal serotonergic signaling and altered expression of genes known to be involved in synaptic long-term potentiation in the striatum (Clarke et al. 2013; Diaz Heijtz et al. 2011; van Opstal and Bordenstein 2015). As a result of neural differences, these mice are less anxious and display increased locomotor activity than specific pathogen-free (SPF) control mice (Diaz Heijtz et al. 2011). The presence of an intact microbiome is, thus, an essential requirement for normal development.

Specific microbes have been found to regulate host gene expression. For example, in cultured human cells, lipopolysaccharide (LPS) from *Escherichia coli* and other proteobacteria caused an inflammatory response by activating toll-like receptor 4 (TLR4), leading to a gene expression cascade of innate immune pathways (Rallabhandi et al. 2008),. Microarray analysis in mice showed that ~ 700 host intestinal genes were differentially expressed between GF mice and SPF mice (Cresci et al. 2010; Liu et al. 2016), which are free of specific pathogens determined from routine testing but are not GF and, thus, harbor a microbiome. The latter showed that bacterial recolonization of the intestinal tract of GF mice reversed some of these gene expression changes. In a different experiment, FMT of germ-free C3H mice, which harbor a mutation in the *Tlr4* gene, with C57BL/10 feces resulted in 202 genes differing more than twofold in expression (Brodziak et al. 2013). The behavioral abnormalities of GF mice related to anxiety were corrected when they were given an SPF microbiome (Cresci et al. 2010; Liu et al.). Similarly, in another mouse experiment, the depletion of the gut microbiome through antibiotic treatment caused cognitive impairment, accompanied by changes in the expression of cognition-relevant signaling molecules in specific regions of the brain (Frohlich et al. 2016). These observations support a host–microbiome interaction model where the microbiome alone can independently modulate the expression of host genes and subsequently affect a phenotype.

There is growing evidence that the effects of the microbiome on host gene expression are modulated through the epigenome. The epigenome is the chemical modifications to DNA and histone proteins that regulate the expression of genes and is thought to be regulated in part by the metabolome (Krautkramer et al. 2017). The metabolome is the collection of metabolites produced during metabolism and includes those metabolites produced by the gut microbiome. Thus, the metabolome can be thought of as the functional intermediate between the microbiome and host gene expression (reviewed in (Krautkramer et al. 2021)). Short-chain fatty acids (SCFAs) produced by the microbiome regulate host defenses and the immune system through epigenetic control by inhibiting histone deacetylation. Butyrate, a known histone deacetylation inhibitor (Davie 2003; Wu et al. 2020), produced by commensal microbes such as Clostridia has been shown to induce regulatory T cell development by enhancing histone H3 acetylation (Furusawa et al. 2013). The gut microbiome induces epigenetics changes of all types, DNA methylation, histone modification and regulation by non-coding RNA to control gene expression of the host.

### Opportunities and considerations in the use of animal models to study host genetic-microbiome interactions

Animal studies allow researchers to have exquisite control over the environment of their animals. Each research location determines what the SPF health status of their vivarium will be. The health status corresponds to which microbes are excluded, through routine testing, from being present within a mouse colony. This variation in SPF status across vivaria has inadvertently enabled researchers to determine that the phenotype of some inbred strains of mice vary based upon the presence of specific microbes. One such example is diabetes, a condition that is present in NOD/ShiLtJt mice. In this well-characterized type 1 diabetes (T1D) model, diabetes develops in young (3–5 week-old) mice as autoreactive T-cells destroy the insulin-producing beta cells in the pancreas. Researchers using this model in vivaria that screen for fewer pathogens (lower SPF status) noticed a decreased incidence of T1D in their NOD/ShiLtJt mice (Pozzilli et al. 1993). This was due to the presence of segmented filamentous bacteria class of microbiota in their facility. The segmented filamentous bacteria class of microbiota is known to influence the severity of the autoimmune response by triggering a counter Th_17_ immune response that decreases the autoreactivity and protects the mice from developing T1D (Ivanov et al. 2009). This same mouse model displayed decreased T1D when given acidified drinking water, which significantly altered the microbiome, specifically levels of Actinobacteria, Proteobacteria, and Firmicutes, similarly decreasing the Th_17_ mediated autoreactivity (Wolf et al. 2014). Thus, the control researchers have over the environment of animals that has provided various, sometimes unexpected insights.

A recent commentary emphasizes the importance of “*Knowing your model and its microbiota*” (Perry et al. 2020). The example that best exemplifies this thesis is the finding that the gut microbiome is causative for a phenotypic change of a mutant transformation-related protein 53 (*Trp53)* to switch from tumor suppressor to oncogene in the context of a genetic model for intestinal cancer (*Csnk1a1*^*tm1*.1Ybn^) (Kadosh et al. 2020). This change was due to the presence of a microbiome-derived metabolite, gallic acid, which abolishes the tumor-suppressive nature of the mutation only in a region of the distal gut where the gallic acid-producing microbe is present. This is an example of an interaction between host genotype (mutant Trp53) and microbiome (gallic acid-producing microbe) that creates an altered phenotype (malignancy). Taken together, a comprehensive view that includes both host genetics and microbiome composition is critical to fully understand the relationship between genes and the manifestation of the disease (van Opstal and Bordenstein 2015).

### Experiments to dissect the cause-and-effect relationships of the host and microbiome

A major challenge for understanding host–microbiome interactions is determining causality of an altered phenotype due to a genetic alteration. As illustrated in Fig. 1, variation in the host genome can alter a phenotype in multiple ways. The ability to transfer a specific phenotype from donor to recipients in FMT experiments is one way to distinguish between the altered microbiome being causative of the disease phenotype or the altered microbiome being a subsequent manifestation of the disease processes. One example is the case of the *NOD2* gene, which is associated with both IBD and an altered microbiome (Frank et al. 2011; Rehman et al. 2011). Wild-type mice were transplanted with fecal material from the *NOD2* knock-out mouse, and the recipient mice developed features of IBD seen in the *NOD2* fecal donor mice (Couturier-Maillard et al. 2013). The transfer of disease phenotype by the transfer of the microbiome suggests that the genetic variant alters the microbiome directly, which results in an altered phenotype (Fig. 1, Pathway III). Similarly, we tested causality using the BKS.Cg-Dock7^m^ + / + Lepr^db^/J mouse, a model of leptin deficiency, resulting in obesity, type-2 diabetes, and abnormal sleep patterns in the form of altered sleep–wake regulation (Laposky et al. 2008). This strain possesses various microbes that are absent in their wild-type littermates (Geurts et al. 2011). We found that treatment of these mice with antibiotics resulted in the restoration of sleep behaviors, suggesting that the genetic variant altered the microbiome, which was responsible for the phenotype (Fig. 1, Pathway III). Additional experimentation is needed to identify the microbe or metabolites involved and the mechanisms through which the microbiome controls sleep behavior. These studies highlight how FMT can be used to define cause-and-effect relationships between microbiome composition and host phenotype.

APP/PS1transgenic mice (Tg), a well-established neurodegenerative model of Alzheimer's disease, are associated with microbiome shifts over time (Bauerl et al. 2018). The microbiomes of wild-type versus. Tg mice begin to diverge at six months of age, with the Tg mice having an increase in the genus *Sutterella* which is concurrent with the time when the animals develop *β*-amyloid deposits in the brain. By 24 months of age, the microbiome of the Tg mice is enriched with *Erysipelotrichaceae*, a known inflammation-related microbe. In this case, it is not yet clear what the status of causality is (Fig. 1, Pathways I, II, or III). Experimental observations such as these make causal experimentation such as FMT a priori to determine to what extent the AD-associated changes accelerate the AD pathology, and if so, whether microbiome-mediated interventions might alter AD pathology.

### Important next steps and priorities

The human and mouse microbiomes have been described as qualitatively alike but quantitatively different (Krych et al. 2013) in that they each include a qualitatively similar core of specific phyla but the abundance of specific phyla and species differ. In contrast to the microbiome, the DNA sequence in the protein-coding regions of the mouse and human genomes is 85% identical (Mouse Genome Sequencing et al. 2002). Because of this genomic conservation, the mouse has an extensive history of being used for understanding the genetics of human health and disease (Hedrich 2012); with emerging populations capturing the genetic diversity equivalent to what is seen in the human population (Saul et al. 2019). The mouse microbiome can be studied in these diverse mouse populations in a controlled laboratory environment, enabling the detection of loci involved in microbial abundance. Modern genomic engineering techniques such as CRISPR/Cas9 make the production of genetic knock-out mice and transgenic mice, a routine procedure. Once loci are identified, they can be validated using these mouse genetic engineering techniques to determine causality.

In order to take the next step and perform necessary causal experimentation, additional resources are needed, and technological barriers need to be addressed. A key limitation to understanding the microbiome of mice through 16S sequencing is the lack of diversity of sequenced microbiomes in the reference database. Much of the 16S reference database sequence comes from human samples which have a different microbiome composition than mice. The C57BL/6 J and *Lep*^*ob*^ microbiomes have been characterized by two groups (Liu et al. 2020; Suez et al. 2014), producing reference WGS or 16S data as well as creating a biobank of 126 species, represented by 244 bacterial strains. This effort included 77 new species being identified. Other groups have addressed microbiome diversity by cataloging the microbiome of six different inbred strains of mice from various institutions that were fed a variety of different diets (Xiao et al. 2015), thereby producing additional mouse-centric 16S data for the databases. The German Mouse Intestinal Bacterial Collection has sequenced the 16S of microbes from wild mice (Lagkouvardos et al. 2016), and made 104 cultural bacterial strains available on their website (www.dsmz.de/miBC). We are currently working with the Diversity Outbred (J:DO) mouse population (Svenson et al. 2012) and routinely find that 30–50% of our 16S sequences are not in databases and/or represent new taxa that have not been phylogenetically placed and, thus, are not classified. 16S sequencing has inherent limitations due to copy number variation, as well as not being ideal to detect some microbial groups. As the field is adopting whole-genome sequencing in place of 16S analysis, we have undertaken whole-genome shotgun-sequencing on a subset of mouse samples from a DO cohort and have assembled over 500 new genomes (unpublished). Other recent studies have produced larger sets of new genomes from mouse microbiomes (Lagkouvardos et al. 2016; Liu et al. 2020; Suez et al. 2014; Xiao et al. 2015).

Cataloging the existence of microbes is only step one. Having the microbes isolated to perform inoculation studies in vivo is an ideal intervention for causality studies. One challenge to this approach is the fact that numerous microbes are not able to be cultured in the laboratory. For other microbes, the strains available at microbial stock centers (such as the American Type Culture Collection) may not be the same as the commensal that are found in the mouse gut. One way that unculturable microbes can be studied is as part of the microbial ecosystem through fecal microbiome transplantations. Other approaches to address the unculturable bacteria include antibiotic ablations or metabolite identification studies. The identification of the metabolites causing the phenotypes circumvents the need to culture the microbes all together.

The host genetic control of the microbiome is not just limited to the taxonomic level but also at the taxa-independent metabolite level. Some metabolites are made solely by microbes (e.g., butyrate), and others that are made by the host can also be made by microbes (e.g., serotonin). Instead of adding the microbe back, the microbiome can be removed, or the metabolic product of the microbe added back to the mice. A single microbe may not be responsible for a phenotype; rather, it may be caused by a group of microbes, some of which are depleted and others overabundant in causing a specific biological trait. Ultimately the microbiome is easier to manipulate (e.g., antibiotics, probiotics, prebiotics) than genome manipulation, and metabolic supplementation is even easier for the treatment of disease. These developments open the door to new therapeutic and diagnostic approaches.

## Conclusions

The past two decades have produced many clues as to how the gut microbiome composition is affected by host genetics. These have come from the human twins, genetic association studies and GWAS, and numerous mouse QTL and gene knock-out studies; however, our overall understanding of the role of the host in the regulation of microbiome composition is in its infancy. While this review focuses on the genetics of the host, the diet and environment of the host are well-known sources of variation of microbiome composition. The superb control of the genetics and environment of laboratory animals within an experiment enables scientists to untangle this mystery. Through the successful design and execution of causal experiments using a controlled intervention such as a genetic knock-out, FMT, specific microbial inoculation, germ-free host, or antibiotic ablation, we will more fully understand the mechanisms controlling the diversity and composition of our bacterial symbionts and commensals. As more mechanisms of host–microbial interactions and the causal relations of microbes and their metabolites to disease become better understood, the development of advanced therapeutic approaches informed by the microbiome becomes a reality.

## Supplementary Information

Below is the link to the electronic supplementary material.

Supplementary file1 (XLSX 28 kb)
